# Removal of Paralytic Shellfish Toxins by Probiotic Lactic Acid Bacteria

**DOI:** 10.3390/toxins6072127

**Published:** 2014-07-18

**Authors:** Mari Vasama, Himanshu Kumar, Seppo Salminen, Carolyn A. Haskard

**Affiliations:** 1 Functional Foods Forum, University of Turku, 20014 Turku, Finland; E-Mails: mari.vasama@gmail.com (M.V.); sepsal@utu.fi (S.S.); 2 Australian Water Quality Centre, Private Mail Bag 3, Salisbury, SA 5108, Australia; E-Mail: carolyn.haskard@csiro.au; 3 School of Pharmacy and Medical Sciences, University of South Australia, GPO Box 2471, Adelaide, SA 5001, Australia

**Keywords:** paralytic shellfish poisoning, saxitoxin, lactobacillus

## Abstract

Paralytic shellfish toxins (PSTs) are non-protein neurotoxins produced by saltwater dinoflagellates and freshwater cyanobacteria. The ability of *Lactobacillus rhamnosus* strains GG and LC-705 (in viable and non-viable forms) to remove PSTs (saxitoxin (STX), neosaxitoxin (neoSTX), gonyautoxins 2 and 3 (GTX2/3), C-toxins 1 and 2 (C1/2)) from neutral and acidic solution (pH 7.3 and 2) was examined using HPLC. Binding decreased in the order of STX ~ neoSTX > C2 > GTX3 > GTX2 > C1. Removal of STX and neoSTX (77%–97.2%) was significantly greater than removal of GTX3 and C2 (33.3%–49.7%). There were no significant differences in toxin removal capacity between viable and non-viable forms of lactobacilli, which suggested that binding rather than metabolism is the mechanism of the removal of toxins. In general, binding was not affected by the presence of other organic molecules in solution. Importantly, this is the first study to demonstrate the ability of specific probiotic lactic bacteria to remove PSTs, particularly the most toxic PST-STX, from solution. Further, these results warrant thorough screening and assessment of safe and beneficial microbes for their usefulness in the seafood and water industries and their effectiveness *in vivo*.

## 1. Introduction

**Figure 1:**
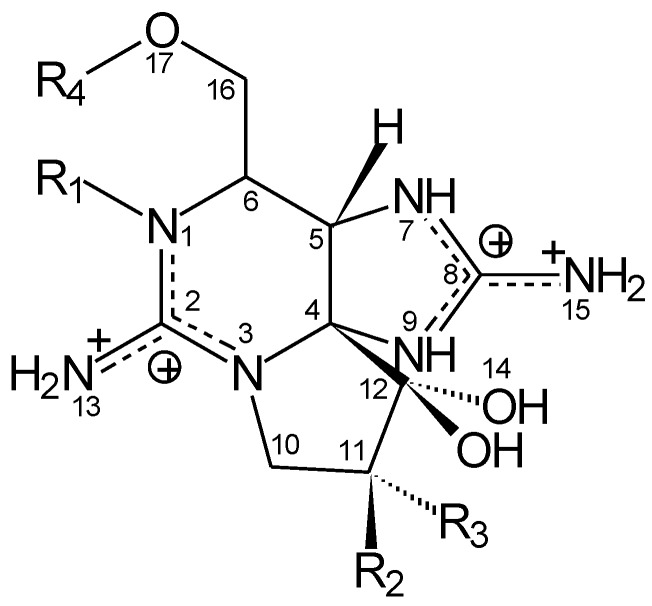
Paralytic shellfish toxins (PST) are a group of water-soluble carbamate alkaloid neurotoxins exhibiting a range of toxicities, which are either non-sulfated (saxitoxin (STX), neo-STX), singly sulfated (gonyautoxins (GTX)) or doubly sulfated (C-toxins) at various positions indicated by R_1–4_.

Paralytic shellfish toxins (PSTs) belong to a family of neurotoxins produced by freshwater cyanobacteria (blue-green algae) [1] and marine dinoflagellates (red tide) [2,3]. Chemically, PSTs are a group of water-soluble carbamate alkaloids, which are either non-sulfated (saxitoxin (STX), neo-STX), singly-sulfated (gonyautoxins (GTX)) or doubly-sulfated (C-toxins) (Figure 1).

**Table:** 

PST	R_1_	R_2_	R_3_	R_4_	Net Charge	Relative Toxicity
STX	H	H	H	CONH_2_	2	1
neoSTX	OH	H	H	CONH_2_	2	0.924
GTX2	H	H	OSO_3_^−^	CONH_2_	1	0.359
GTX3	H	OSO_3_^−^	H	CONH_2_	1	0.638
C1 (epiGTX8)	H	H	OSO_3_^−^	CONHSO_3_^−^	0	0.006
C2 (GTX8)	H	OSO_3_^−^	H	CONHSO_3_^−^	0	0.096

Ingestion of adequate amounts of PST leads to paralytic shellfish poisoning. The toxins block the influx of sodium ions in sodium channels, subsequently restricting signal transmission along neurons and causing death from respiratory failure [1]. PSTs appear to be more hazardous to terrestrial mammals than to aquatic biota [1], and they represent a significant health risk for humans, cattle, sheep, pigs, dogs and wildlife. In mice, LD50 of STX was found to be 260 μg/kg by the oral route [4], but the effect of long-term, low-level exposure to PSTs is unknown.

In most cases, the level of PSTs in contaminated water itself is insufficient to cause paralytic shellfish poisoning. However, exposure to higher levels of PSTs can arise from the consumption of contaminated seafood (including mussels and oysters), algal dietary supplements and toxin-producing cyanobacterial cells. During cyanobacterial blooms, livestock mortalities have occurred after ingestion of water containing toxic cyanobacteria [5]. The incidence of human paralytic shellfish poisoning has also been increasing, causing a severe public health problem, as well as problems for the food industry. For instance, STXs has been reported in drinking water in Australia and Brazil [6]. Seafood regulations (EU directive 91/492/EEC) set the maximum acceptable limit of PSTs in shellfish at 80 µg STX equivalents 100 g^−1^ tissue [7].

There is no known antidote or effective medical treatment available for paralytic shellfish poisoning [8]. Methods of removing PSTs during the drinking water treatment process have been studied, but each has limitations [9]. An efficient cost-effective means of inactivating or removing these dietary toxins, either *in vitro* or *in vivo*, is needed in order to reduce human fatalities and livestock deaths, especially in developing countries.

Specific probiotic lactic acid bacteria have previously been shown to inactivate potent food-borne toxins, including aflatoxin B1 and other mycotoxins, as well as mutagenic dietary heterocyclic aromatic amines, to varying degrees [9,10]. Lactic acid bacteria are important natural constituents of the human gastrointestinal tract and are utilized widely in the food industry. Some are classified as probiotics; bacteria that have confirmed positive effects on health. Specific probiotics have been shown to be effective in treating and preventing different types of diarrhea and allergies, as well as stimulating the immune system [11]. In this study, the effectiveness of two probiotic lactic acid bacteria, *Lactobacillus rhamnosus* strains GG (GG) and LC-705 (LC-705) (in viable and non-viable forms), in removing six PSTs from acidic or neutral solutions, which represent pH variation in the gastrointestinal tract, was investigated using HPLC.

## 2. Results and Discussion

This study was designed to gain information about the PST removal mechanism by both viable and non-viable probiotic lactic acid bacteria. The removal of six specific PSTs in concentrations of 44.8–397.8 μg/L by GG and LC-705 varied significantly between the different toxins (Table 1). The effect of an acidic environment on bacterial removal of PSTs was investigated, as this information is useful for application in acidic foods and also *in vivo*, since both bacteria and PSTs pass through the acidic stomach prior to the intestine.

The highest degree of removal was observed for STX and neoSTX (77%–97.2%). This was significantly higher (*p* < 0.05) than the intermediate removal detected for GTX3 and C2 (33.3%–49.7%), which was, in turn, significantly higher (*p* < 0.001) than the poor removal observed for C1 (7.8%–13.7%). There was no significant difference between the removal of STX from the pure mix and C2 from the extract and between the removal of GTX2 and either C1 or GTX3/C2 from the extract. Removal of PSTs decreased in the order: STX ~ neoSTX > C2 > GTX3 > GTX2 > C1. This is similar to the relative toxicity order (Figure 1), with the exception of C2. Correlations between the three-dimensional structure of PSTs and their level of removal (Table 1) are consistent with the two positively charged guanidinium nitrogens associated with a negatively-charged bacterial surface. There are distinct charge differences between the three classes of PSTs; that is STXs (+2), GTXs (+1) and C-toxins (0) (Figure 1). The present results suggest that electrostatic and hydrophobic interactions may be important in bacterial binding of toxins.

**Table 1:** Removal of paralytic shellfish toxins from solution following incubation (37 °C, 1 h) with *L. rhamnosus* strains GG or LC-705. STX, saxitoxin; neoSTX, neosaxitoxin; GTX, gonyautoxin; C1, C-toxin 1.

Substrate	Concentration (μg/L)	Co-Incubated with	Incubation Buffer pH	% Removed ^a^ by *L. rhamnosus* Strain GG		% Removed ^a^ by *L. rhamnosus* Strain LC-705	
				Viable ^b^	Non-viable ^c^	Viable ^b^	Non-viable ^c^
STX ^d^	120		7.3	79.5 ± 2.4	84.9 ± 1.6	89.9 ± 2.8	85.6 ± 1.0
neoSTX ^e^	120			>80.2 ± 2.4	>97.2 ± 2.1	>77.0 ± 2.4	>91.9 ± 0.9
STX	50.4	Organics ^f^	7.3	81.2 ± 2.1	90.0 ± 0.9	87.6 ± 0.8	91.0 ± 0.9
GTX2	154.2			32.5 ± 2.5	18.8 ± 5.2	27.0 ± 7.6	25.7 ± 3.1
GTX3	44.8			36.0 ± 2.0	39.6 ± 2.1	42.7 ± 3.1	45.1 ± 1.2
C1	397.8			13.7 ± 2.8	9.8 ± 0.5	12.8 ± 0.5	12.6 ± 1.2
C2	228.8			46.1 ± 1.4	47.8 ± 0.4	49.7 ± 0.6	47.8 ± 1.5
STX	50.4	Organics ^f^	2.0	66.2 ± 1.9	87.3 ± 2.0	74.1 ± 1.0	89.1 ± 0.8
GTX2	154.2			26.3 ± 5.3	30.4 ± 6.6	38.2 ± 10.0	33.4 ± 2.3
GTX3	44.8			37.7 ± 6.8	33.3 ± 7.5	44.7 ± 1.1	44.7 ± 6.0
C1	397.8			11.8 ± 1.4	12.6 ± 0.4	7.8 ± 0.9	12.2 ± 2.6
C2	228.8			38.2 ± 2.3	37.0 ± 2.6	41.7 ± 1.6	43.9 ± 1.3

^a^ Data are expressed as mean ± SD (*n* = 3); ^b^ viable; ^c^ non-viable (boiled in PBS for 1 h); ^d^ STX in the pure mix of STX and neoSTX solution; ^e^ another compound had the same retention time; therefore, values reflect a minimum amount of removal; ^f^ the solution contained multiple paralytic shellfish toxins (PSTs) and at least 10 mg/L dissolved organic carbon extracted from cyanobacterial scum material.

There was no significant difference between the removal by GG and LC-705, nor between removal by viable and non-viable bacteria. Overall, bacterial removal of PSTs was significantly greater in neutral buffer (pH 7.3) than in acidic buffer (pH 2) (*p* < 0.01). Some *L. rhamnosus* strains are reported to have a net negative surface charge at neutral pH [12]. Electrostatic interactions existing between positively-charged PSTs and the negatively-charged bacterial surface at neutral pH would be reduced in an acidic environment. This could explain the greater removal of PSTs observed in neutral solution in comparison with acidic solution. While the level of PST removal generally dropped in acidic solution, it was never less than 60% of the removal achieved at neutral pH. Under acidic conditions, the binding of PSTs must result from other types of interactions, such as hydrogen bonding.

### 2.1. Removal of STX and neoSTX

Removal of the most toxic PSTs—STX and neoSTX—was high and not significantly different. In general, non-viable heat-treated bacteria removed more STX than viable bacteria, and this difference is accentuated in acidic solution. The same trend may occur for neoSTX, but this cannot be ascertained from the data in Table 1. For the viable bacteria, LC-705 appears to remove more STX than GG.

The effect of the presence of other organic molecules on the removal of specific PSTs is shown in Table 1. There was no significant difference between removal of STX from the mix of pure STX and neoSTX only and its removal from the cyanobacterial extract solution containing a range of PSTs and other organic molecules. Hence, overall, the presence of considerably higher concentrations of organic molecules and the presence of multiple PSTs had no significant effect on inhibiting or enhancing the removal of STX. However, the presence of additional organics appeared to enhance the removal of STX by non-viable bacteria.

The concentration of PST in solution can affect the amount of PST removed. Nevertheless, similarities in the removal level of STX at concentrations of 50.4 and 120 μg/L suggest that concentration has a minor impact on the removal levels within this range.

Comparison to previous studies with other substrates can be difficult, as the amount of substrate removed varies with substrate concentration and the bacterial cell counts used [13]. Nevertheless, the percentage removal of STX (50.4–120 μg/L) and neoSTX (120 μg/L) by both non-viable and viable LC-705 and GG are similar to the amounts of the potent hepatic mycotoxin, aflatoxin B_1_ (5 μg/mL), removed by these organisms in similar conditions [13,14]. Removal of the dietary heterocyclic aromatic amine, PhIP (50 μg/mL), is also similar at pH 3, where it is positively charged and has some structural similarity with PSTs [13].

### 2.2. Removal of GTX2, GTX, C1 and C2

Removal of GTXs ranged between 18.8% and 45.1% depending on the bacteria, the incubation buffer pH and the GTX analogue. In most cases, the removal of GTX3 was either greater or not significantly different than GTX2. LC-705 removed more GTX3 than GG in neutral solution. The removal of GTX2 by non-viable GG was considerably less than viable GG, and it appeared to be enhanced by acidic buffer in comparison with the neutral buffer.

In all cases, the removal of C2 was greater than C1. The amount of C1 removed varied from 7.8% to 13.7%, while C2 was removed between 37.0% and 49.7%. Removal of C2 was less effective in acidic buffer in comparison with the neutral buffer.

## 3. Experimental Section

### 3.1. Bacterial Preparation

*Lactobacillus rhamnosus* strains GG (ATCC 53103) and LC-705 (DSM 7061) were obtained as a freeze-dried powder from Valio (Helsinki, Finland) and Ron Hull & Associates (Mt Waverley, Victoria, Australia), respectively. These strains were pre-cultured and then cultured (42 °C, 9% CO_2_) for 24 h in MRS broth. Bacterial pellets were collected by centrifugation (13,000× *g*, 4 °C, 10 min), washed with phosphate buffered saline (PBS; 0.08 M phosphate, pH 7.6) and stained with Syto 9. Bacteria were enumerated by flow cytometry using a FACS Calibur flow cytometer (Becton Dickinson, San Jose, CA, USA). Briefly, cells were counted over a 2-min period, and the corresponding volume of bacterial suspension was used for enumeration. Non-viable bacteria were obtained by heat-treatment (boiled at 95 °C, 1 h) in PBS (2 mL), followed by centrifugation (3210× *g*, RT, 10 min), and the supernatant was removed.

### 3.2. Preparation of Cyanobacterial Extract Containing PSTs

Frozen scum material, collected during a bloom of toxic *Anabaena circinalis* (Coolmunda Dam, Warwick, Queensland, Australia, May 1997), was freeze-thawed three times to lyse the cells. Following centrifugation (16,900× *g*, 4 °C, 30 min), the supernatant was removed and the pellet was resuspended in 0.05 M acetic acid. Following sonication (30 min) and centrifugation (16,900× *g*, 4 °C, 30 min), the supernatants were combined and concentrated (rotary evaporation, 40 °C) to one third their original volume. To the concentrated extract, an equal volume of cold 95% ethanol was added, and the solution allowed to stand on ice for 1 h. After centrifugation (16,900× *g*, 4 °C, 30 min), the supernatant was removed and concentrated (rotary evaporation, 40 °C) to one quarter of its original volume, to remove most of the ethanol. The concentrated extract was then filtered through a glass-fiber filter (GF/C).

Pigment removal was carried out using C18 column chromatography. A methanol slurry of the C18 packing (200 g per 500 mL concentrated extract; Waters bulk packing material, 55–105 µm) was used to produce a bed of approximately 5 cm in depth in a glass chromatography column (10-cm diameter), and the packing was washed by 500 mL of 80%, 60%, 40% and 20% methanol and 1 L water. The concentrated extract was added to the column and drained to the surface of the packing with any eluate being collected. The column was then eluted with water (1 L) and 0.05 M acetic acid (500 mL), the eluates combined and concentrated by rotary evaporation. The concentrations of toxins in the final extract were (mg/L): 0.25 (STX), 0.77 (GTX2), 0.22 (GTX3), 1.99 (C1) and 0.92 (C2). The extract also contained decarbamoyl gonyautoxins (dcGTX2 and dcGTX3), which could not be quantified, due to a lack of standards. The pH of the extract was 3.48, and the dissolved organic carbon content was at least 10 mg/L.

Two working PST solutions were prepared from this extract by dilution with two phosphate buffers (0.01 M, pH 7.3 and pH 2.0) to give final solution concentrations of (μg/L): 50.4 (STX), 154.2 (GTX2), 44.8 (GTX3), 397.8 (C1) and 228.8 (C2).

### 3.3. Preparation of Pure PST Solution

Pure STX and neoSTX were purchased from the Institute for Marine Biosciences (National Research Council, Ottawa, ON, Canada), and stock solutions (14.4 mg/L) were prepared in 0.05 M acetic acid. A working solution of pure PSTs (containing 120 μg/L STX and 120 μg/L neoSTX) was prepared in neutral buffer from these stock solutions.

### 3.4. Sample Preparation

Bacterial pellets in triplicate (10^10^ cfu) were suspended with PST solution (1 mL) and incubated (37 °C, 1 h) with shaking. PST solution was used as a positive control, and the buffer was substituted for PST solution in negative controls. After centrifugation (3210× *g*, RT, 10 min), the supernatants were removed and filtered (0.45 µm PVDF membrane, Pall Corp., Washington, NY, USA), prior to HPLC analysis.

### 3.5. HPLC Analysis

Reverse phase HPLC with post-column derivatization and fluorescence detection was carried out for each of the three classes of PST (STXs, GTXs and C-toxins) by the methods described by Oshima [15]. Samples were injected directly (volume varied between 5 and 200 μL) to achieve detection limits of ≤0.3 ng for each injected PST.

A C8 column (150 mm × 4.6 mm) with a C8 guard cartridge was used for all toxin analyses. The column oven was set to 20 °C for C-toxin and GTX analyses and to 40 °C for STX analyses. A flow rate of 0.8 mL/min was used, and the injection volume varied between 5 and 200 μL. Post column, toxins reacted with oxidant and acid in a Teflon coil (0.5 mm i.d. × 10 m) at 65 °C in a dry air oven. Detection was by fluorescence with excitation and emission wavelengths of 330 and 390 nm, respectively, with a bandwidth of 18 nm. A flow rate of 0.4 mL/min was used for post column reagents. All samples were passed through 0.45 μm filters prior to analysis.

For C-Toxin analyses, the mobile phase was 1 mM tetrabutyl ammonium phosphate (adjusted to pH 6 with 1 M sodium hydroxide). The post column oxidant solution was 7 mM periodate in 50 mM potassium dihydrogen phosphate, adjusted to pH 9.0 with 5 M sodium hydroxide, and the post column acid solution was 0.5 M acetic acid. The run time was 8 min, and the retention times for C1 and C2 were 4.3 and 4.9 min, respectively.

For GTX analyses, the mobile phase consisted of 2 mM sodium heptane sulfonate and 10 mM diammonium hydrogen orthophosphate adjusted to pH 7.1 with phosphoric acid. The post column oxidant solution was 7 mM periodate in 50 mM diammonium hydrogen orthophosphate, adjusted to pH 7.8 with 5 M sodium hydroxide, and the post column acid solution was 0.5 M acetic acid. The run time was 16 min, and the retention times for GTX2 and GTX3 were 12.8 and 10.3 min, respectively.

For STX analyses, the mobile phase consisted of 2 mM sodium heptane sulfonate, 25 mM diammonium hydrogen orthophosphate and 5% acetonitrile, adjusted to pH 7.1 with phosphoric acid. The post column reagents were the same as used for the GTX analyses. The run time was 12 min, and the retention times for STX and neoSTX were 8.3 and 7.0 min, respectively.

### 3.6. Statistical Analysis

Statistical analysis was carried out using PRISM (version 4.0, Graph Pad Software Inc, San Diego, CA, USA). Two-way ANOVAs between each pair of variables suggested that the toxin was responsible for >90% of the total variation. The interaction between the toxin and bacteria accounted for <2% of the variation, as did the interaction between the toxin and buffer. Bacteria, buffer and any interaction between them were not a significant source of variation. Since data for the two buffers used did not pass the normality test, a non-parametric Wilcoxon signed rank test (2-tailed, matched pairs) was used. A one-way ANOVA was carried out between results for the four bacteria, and Tukey’s *post hoc* test was used to determine significant differences. A non-parametric Kruskal–Wallis test was used to compare results for the seven toxins, as the variances were significantly different, and Dunn’s *post hoc* test was used to determine significant differences. Results were considered significantly different at the 95% confidence interval (*p* < 0.05).

## 4. Conclusions

The strong removal of PSTs by non-viable bacteria indicates that PSTs are removed by binding rather than by metabolism. The lack of a significant difference between the ability of non-viable and viable bacteria to remove PSTs suggests viable bacteria also remove PSTs by binding. This is consistent with previous reports for the removal of a range of mycotoxins and other substrates by these specific bacterial strains through binding [10,16,17]. The advantage of non-viable bacteria could be attributed to their varied utility in food applications, water treatment and *in vivo* applications.

Very little is known about how PSTs are absorbed through the mammalian intestinal epithelium; however, altering the intestinal microflora composition with probiotics can reduce the uptake of harmful compounds and prevent sickness and disease [18,19]. This is the first report of the ability of probiotic bacteria to effectively remove PSTs from solution. Further studies are needed to investigate the potential use of these probiotic microbes for industrial application or health benefits.
